# Obatoclax is a direct and potent antagonist of membrane-restricted Mcl-1 and is synthetic lethal with treatment that induces Bim

**DOI:** 10.1186/s12885-015-1582-5

**Published:** 2015-08-01

**Authors:** Mai Nguyen, Regina Cencic, Franziska Ertel, Cynthia Bernier, Jerry Pelletier, Anne Roulston, John R. Silvius, Gordon C. Shore

**Affiliations:** 1 Department of Biochemistry, McGill University, Montreal, Québec Canada; 2 Goodman Cancer Research Center, McGill University, Montreal, Québec Canada

## Abstract

**Background:**

Obatoclax is a clinical stage drug candidate that has been proposed to target and inhibit prosurvival members of the Bcl-2 family, and thereby contribute to cancer cell lethality. The insolubility of this compound, however, has precluded the use of many classical drug-target interaction assays for its study. Thus, a direct demonstration of the proposed mechanism of action, and preferences for individual Bcl-2 family members, remain to be established.

**Methods:**

Employing modified proteins and lipids, we recapitulated the constitutive association and topology of mitochondrial outer membrane Mcl-1 and Bak in synthetic large unilamellar liposomes, and measured bakdependent bilayer permeability. Additionally, cellular and tumor models, dependent on Mcl-1 for survival, were employed.

**Results:**

We show that regulation of bilayer permeabilization by the tBid – Mcl-1 - Bak axis closely resemblesthe tBid - Bcl-XL - Bax model. Obatoclax rapidly and completely partitioned into liposomal lipid but also rapidly exchanged between liposome particles. In this system, obatoclax was found to be a direct and potent antagonist of liposome-bound Mcl-1 but not of liposome-bound Bcl-XL, and did not directly influence Bak. A 2.5 molar excess of obatoclax relative to Mcl-1 overcame Mcl-1-mediated inhibition of tBid-Bak activation. Similar results were found for induction of Bak oligomers by Bim. Obatoclax exhibited potent lethality in a cellmodel dependent on Mcl-1 for viability but not in cells dependent on Bcl-XL. Molecular modeling predicts that the 3-methoxy moiety of obatoclax penetrates into the P2 pocket of the BH3 binding site of Mcl-1. A desmethoxy derivative of obatoclax failed to inhibit Mcl-1 in proteoliposomes and did not kill cells whose survival depends on Mcl-1. Systemic treatment of mice bearing Tsc2^+^^*/*^^-^ Em-myc lymphomas (whose cells depend on Mcl-1 for survival) with obatoclax conferred a survival advantage compared to vehicle alone (median 31 days vs 22 days, respectively; *p*=0.003). In an Akt-lymphoma mouse model, the anti-tumor effects of obatoclax synergized with doxorubicin. Finally, treatment of the multiple myeloma KMS11 cell model (dependent on Mcl-1 for survival) with dexamethasone induced Bim and Bim-dependent lethality. As predicted for an Mcl-1 antagonist, obatoclax and dexamethasone were synergistic in this model.

**Conclusions:**

Taken together, these findings indicate that obatoclax is a potent antagonist of membranerestricted Mcl-1. Obatoclax represents an attractive chemical series to generate second generation Mcl-1 inhibitors.

## Background

Evasion of apoptosis is a hallmark of most cancers and can be achieved through dysregulated expression of the Bcl-2 protein family. Moreover, the changes in Bcl-2 family members that help promote cell survival in the face of oncogenic signaling can also contribute to the resistance to many treatment therapies [1, 2]. The family is comprised of the pro-survival members Mcl-1, Bcl-2, Bcl-XL, Bcl-b, Bfl-1/A1and Bcl-w; the pro-apoptotic effector proteins Bax and Bak; and the pro-apoptotic transducers (tBid, Puma, Bim, Bad, Bik, Noxa, Hrk and Bmf). The transducers link upstream stress signaling to the induction of mitochondrial outer membrane permeabilization (MOMP) by Bax and Bak, resulting in caspase activation and apoptosis. All of the transducers (called BH3-only proteins), once activated to allow exposure of their BH3 helix, can bind and inhibit one or more of the pro-survival members with differing specificities, whereas three (Bim, Puma, tBid) can also interact transiently with Bax and Bak, seeding a complex process of protein oligomerization, transmembrane pore formation and MOMP, resulting in the release of caspase activators from the intermembrane space [3–5]. MOMP is regulated by the strictly binary and competing protein-protein interactions that can occur between the pro-apoptotic and the pro-survival family members, in which the hydrophobic face of the exposed BH3 helix of activated pro-apoptotic members makes contact with complementary binding pockets (P1-P4) located in a surface groove of the pro-survival members. Thus, in the face of excess pro-survival members, activated Bim, Puma, tBid, Bax and Bak with exposed BH3 helices are sequestered and restrained from executing MOMP; but those BH3-only proteins such as Bad and Noxa, which interact only with specific pro-survival members and not with Bax or Bak, have the potential to compete with these interactions and “adjust” the Bcl-2 rheostat to now favor the full activation of Bax and Bak, resulting in pore formation [6–9]. And this in fact has formed the basis for developing peptide or small molecule mimetics of these “sensitizing” BH3-only proteins, as a way to therapeutically adjust the Bcl-2 rheostat to favor cell death instead of cell survival in the cancer setting [5, 10].

Studies of the tBid - Bcl-XL-Bax axis in reconstituted synthetic proteoliposomes have shown that the lipid bilayer plays an active role in the early events and regulation of Bax pore formation and MOMP, in part by contributing to the essential conformational changes that take place in Bcl-XL and Bax in response to tBid [11, 12]. Both in cells and in reconstituted proteoliposomes, tBid also triggers the migration of Bcl-XL and Bax from a primarily membrane-free location to one that is membrane-bound [12]. This is in contrast to Mcl-1 and Bak, which are constitutively anchored at the mitochondrial outer membrane whether or not a cell is stressed [1]. The extent to which this constitutive location permits the tBid - Mcl-1 - Bak axis to deviate from the tBid - Bcl-XL - Bax model is not completely understood but events for Bax and Bak at the membrane surface are quite similar [9, 13].

The clinical stage small molecule obatoclax is a Mcl-1 antagonist [14] that is predicted by *in silico* docking to occupy the P1 and P2 BH3 binding sites in Mcl-1 [15]. Its hydrophobic characteristics make it insoluble in aqueous media, which has precluded valid analyses of mechanism of action by many standard biochemical approaches, despite such data being reported [16]. Thus, it remains to be proven if this agent can directly bind and inhibit Mcl-1 protein as opposed to influencing Mcl-1 activity in cells or in isolated mitochondria by indirect means. In cells, obatoclax is strongly membrane associated but can be redirected to a distinct membrane site dependent upon the presence of excess, ectopic membrane-anchored Bcl-2 at that site [14]. In the case of Mcl-1, concentration of obatoclax at its native membrane location(s) could provide an advantage in promoting access to this constitutive membrane-associated protein. Here, we characterize the dynamic interactions of obatoclax with lipid bilayers. Employing Mcl-1 and Bak constitutively anchored to reconstituted proteolipsomes, we show for the first time that obatoclax is a direct and potent inhibitor of Mcl-1, overcoming Mcl-1’s ability to restrain tBid-induced activation of Bak. Additionally, obatoclax is shown to cooperate with the induction of Bim as a synthetic lethal partner to drive cell death.

## Methods

### Antibodies

The following antibodies directed to human proteins were used: Polyclonal rabbit antiBim (recognizing primarily BimEL in this study) (Stressgene, AAP-330), polyclonal rabbit antiMcl-1 (Stressgene, AAP-240), monoclonal hamster antiBcl-2 (BD, 551052), rabbit antiBcl-XL (produced in-house), polyclonal rabbit antiBax(N-20) (Santa Cruz, sc-493-G), rabbit polyclonal antiBak (Upstate, 06–536), monoclonal mouse antiActin (ICN Biomedicals, Inc, 69100), and monoclonal mouse antiGAPDH (Abcam, 9484).

### Liposome reagents

Egg phosphatidylcholine (PC), egg phosphatidylethanolamine (PE), dioleoylphosphatidylserine (PS), bovine liver phosphatidylinositol (PI), bovine heart cardiolipin (CL) and DOGS-NTA-Ni were purchased from Avanti Polar Lipids Inc. N-(4-maleimidobutyroyl)-PEG_3_-POPE (Mal-PEG_3_-PE) was synthesized as described previously [17]. Calcein was purchased from Sigma and purified on Sephadex LH-20 [18]. The tris-(nitrilotriacetic acid)-modified lipid DOD-tris-NTA was prepared as described [19].

### Proteoliposomes

cDNAs encoding N**-**Flag-human Bak C14A, C166A, ΔC186 − 211 and N-Flag-human Mcl-1 C16A, C286A, Δ328-361, each tagged at the carboxyl terminal with hexa-His tag and a terminal Cys, were constructed using standard recombinant techniques, and the constructs sequence verified. The cDNAs were cloned into pET151 vector and introduced into BL21Star bacterial cells. Recombinant proteins were purified from the bacterial soluble extracts using Ni^2+^-NTA resin as described [20]. For the preparation of large unilamellar liposomes (LUVs), a basic mixture of lipids composed of PC:PE:PS:PI:CL in a weight ratio of 46:25:11:8 was used. In order to anchor recombinant Bak and Mcl-1, 2 mol % Mal-PEG_3_-PE and 1 mol % DOGS-NTA-Ni was also included. LUVs were generated by mixing the lipids in 100 mM KCl, 10 mM HEPES, pH 7.0 followed by extrusion through 0.2 μm polycarbonate filters as described previously [17]. Where indicated, calcein (50 mM, plus 10 mM HEPES and KCl to an osmolarity equal to that of 100 mM KCl/10 mM HEPES) was encapsulated in LUV as described [17]; unencapsulated calcein was then removed on a Sepharose CL-4B column. Binding of Mcl-1 and Bak recombinant proteins to the liposomes was carried out as described [17].

### Calcein release from proteoliposomes

LUV (2 mg lipid/ml) incorporating 1.5 mol% DOD-tris-NTA in the lipid component and encapsulating 50 mM calcein were charged with 5 mM NiCl_2_ for 15 min at room temperature; unbound NiCl_2_ was then removed by passing through Sephadex G-75. The nickel-charged liposomes (0.15 mg lipid/ml) were incubated with proteins (at the indicated amounts) with or without obatoclax (SelleckChem Inc.) or des-methoxy obatoclax (ZCS Inc) or Noxa BH3 peptide (BioPeptide Co., sequence: CAELEVECATQLRRFGDKLNFRQKL-OH) at room temperature for 1 h. Proteoliposomes were recovered by centrifugation at 80 K rpm for 15 min at 4 °C using a Beckman Coulter Optima Max Ultracentrifuge and resuspended to the same pre-centrifugation volume. 20 μl were diluted with 100 μl of 100 mM KCl, 10 mM Hepes pH 7.0, 0.2 mM EDTA, 50 μM DTPA in a Corning 96-well black flat bottom plate. Baseline fluorescence (F_0_) was read in a Tecan Safire at λ_ex_ 488 nm and λ_em_ 525 nm for 5 min after which time 40 nM tBid (recombinant human Caspase-8-cleaved BID, R&D Systems) was added and further readings (F) were obtained. To determine the total potential fluorescence (F_total_), 3 μl of 1 % Triton X-100 was added to the well and one reading was taken. tBid-mediated release of calcein was expressed as F – F_0_/ F_total_.

### Proteoliposome chemical crosslinking

Liposomes containing 2 mol% Mal-PEG_3_-PE and 1 mol % DOGS-NTA-Ni (2 mg lipid/ml) were incubated with recombinant Bak with or without recombinant Mcl-1 at the indicated amounts in 100 μl 100 mM KCl, 10 mM Hepes pH 7.0, for 1 h at room temperature in the presence or absence of obatoclax or Noxa or Bim (Biopeptide Co., sequence: MRPEIWIAQELRRIGDEFNAYYAR-OH) BH3 peptide. Proteoliposomes were recovered by centrifugation at 80 K rpm for 15 min at 4 °C using a Beckman Coulter Optima Max Ultracentrifuge. Proteoliposomes were resuspended in the same pre-centrifugation volume and the primary amine cross-linker BS^3^ (bis(sulfosuccinimidyl)suberate; Pierce) or vehicle (DMSO) was added. Cross-linking was carried out at room temperature for 1 h. Reactions were quenched with 0.1 M Tris pH 9.0 and analyzed by 4–16 % acrylamide SDS-PAGE and immunoblotting.

### Cells and treatments

KMS-11 and TE671 cells were grown in RPMI-1640 supplemented with 10 % FBS, 10 mM Hepes and 10 mM sodium pyruvate. For dexamethasone treatment, KMS-11 cells were seeded at a density of 2.5 × 10^5^ cells per well in 12-well plates (Costar) and treated for 48 h with drug or vehicle (DMSO) in the presence or absence of 40 μM zVAD-fmk (Biovision). Cell viability was determined using Cell-Titer Glo (Promega) according to the manufacturer’s protocol. Data are expressed as the mean of triplicates with SEM after normalizing to control DMSO. Total cell lysate was analyzed by immunoblotting. For caspase activation, 25 μg of total cell lysate (in 100 μl of 50 mM Hepes pH 7.4, 5 mM EDTA, 1 % Triton X-100) was incubated with 50 μM DEVD-AMC, 2 mM DTT for 30 min at 37 °C after which time the reaction was diluted 10 fold with water and read in a Tecan Safire at λ_ex_ 380 nm and λ_em_ 450 nm.

### siRNA knockdown

KMS-11 cells were plated at 2.5 × 10^5^ cells per well in 12-well plates and immediately subjected to siRNA knockdown for 24 h. Cells were transfected with 10 nM of the indicated targeted siRNA or control (non-targeting scrambled) siRNA (siCtl) (Ambion life technologies siRNA negative control product number: AM4611; siRNA Bim ID# s195011) using lipofectamine2000. When transfected with a combination of siRNAs, 10 nM of each siRNA was used. TE671 cells were reverse transfected with equimolar of control siRNA (Dharmacon D-001810-10-50), siRNA to human Bcl-XL (Dharmacon L-003458-00-0050) or human MCL-1 (Dharmacon L-004501-00-0050) for the final concentration of 30 nM total siRNA per well of 12-well dish using RNAiMax (Invitrogen). Cells were collected 48-hour post transfection and subjected to cell death and immunoblotting analyses. For siRNA and obatoclax combination, cells were transfected with siRNA as described for 24 h followed by 48-hour treatment of vehicle (DMSO) or 200 nM obatoclax.

### shRNA knockdown

Tsc2^+/−^*Eμ-Myc* lymphomas were maintained in B-cell media (45 % DMEM, 45 % IMDM, 55 μM β-mercaptoethanol and 10 % fetal bovine serum) on γ-irradiated *Arf*^*−/−*^ MEF feeder layers. Retroviral packaging was performed using ecotropic Phoenix cells according to established protocols (http://web.stanford.edu/group/nolan/_OldWebsite/protocols/pro_helper_dep.html). Tsc2^+/−^*Eμ-Myc* lymphomas were infected with MLS retrovirus expressing shFLuc.1309 as neutral control [21] or shMcl-1.1334 [22]. The amount of GFP^+^ cells was determined 12 h after transduction (t = 0) and again 15 h later by flow cytometry using a Guava EasyCyte HT FACScan instrument and Guava ExpressPro software (Millipore).

### Drug combination studies

KMS-11 cells were plated at 2000 cells/well in triplicate into 96 well plates. Dexamethasone (dex) was added at low doses up to 20 nM for 72 h prior to addition of a dose range of obatoclax for 48 h. Cell viability was assessed using the Cell-Titer Glo assay (Promega). IC_50_ values for dose response curves of obatoclax at each concentration of dex were determined by normalizing the obatoclax-only treated samples to 100 % viability and then curve fitting the obatoclax dose range in the presence of dex using non-linear regression in Prism 5.0 (Graphpad). The combination index (CI) at different concentrations of dexamethasone was calculated using COMPUSYN V.1.0 software according to the original method from Chou and Talalay [23].

### Murine lymphoma models

Treatment studies and analyses were performed on 6–8 week old C57BL/6 mice that had been injected intravenously with 10^6^ Tsc2^+/−^*Eμ-Myc* or *Eμ-Myc/*(myr)Akt lymphoma cells, according to methodology reported previously [24, 25]. Palpable tumors refers to the earliest manual detection of enlarged lymph nodes; complete response refers to the lack of palpable tumors in response to treatment; and relapse refers to the reappearance of palpable tumors. Treatments were either started two days after tumor cell injection (for overall survival studies) or when tumors were palpable (for tumor free survival studies). Obatoclax was administered in 1:1 cremaphor : EtOH (9.25 % each)/5.25 % D_2_O/6.75 % DMSO and mice were treated daily for 5 days (10 mg/kg on days 1, 4 and 5 and 5 mg/kg on days 2 and 3) via intraperitoneal (i.p.) injection. For combination studies, mice were treated with obatoclax for five consecutive days, with doxorubicin delivered once on the second day (10 mg/kg in ddH_2_O). Mice were monitored daily for tumor burden.

Tumor-free survival was defined as time between remission and reappearance of tumors. The experimental endpoint for overall survival is defined by the McGill University Faculty of Medicine Animal Care Committee, which uses the body condition score (BCS) method (United Kingdom Co-ordinating Committee on Cancer Research) (UKCCCR). Guidelines for the welfare of animals in experimental neoplasia (second edition). Br J Cancer 1998; **77**: 1–10 http://cancerres.aacrjournals.org/content/72/3/747.long - ref-15). We used a BCS < 2 which includes decreased exploratory behaviour, reluctance to move, pronounced hunched posture, and moderate to severe dehydration. All animal studies were approved by the McGill University Faculty of Medicine Animal Care Committee. Data was analyzed using the log-rank (Mantel-Cox) test using SigmaStat software and is presented in Kaplan-Meier format.

## Results and discussion

**Fig. 1:**
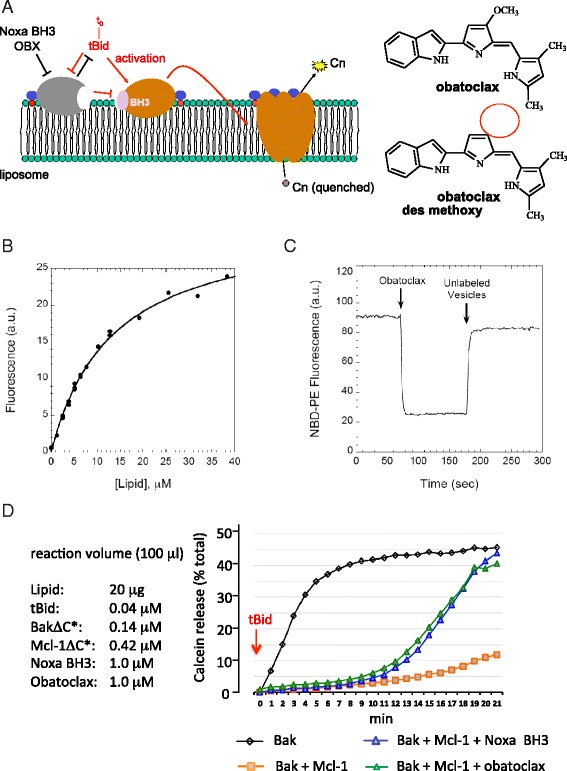
Noxa BH3 peptide and membrane-restricted obatoclax (OBX) directly antagonize the ability of Mcl-1 to inhibit Bak-mediated calcein release from proteoliposomes. **a** Left. Model for the regulation of liposome bilayer permeabilization by the tBid-Bak-Mcl-1 axis. Membrane anchoring of Mcl-1 and Bak is achieved by replacing their C-terminal TM segments with chemical functionalities (blue circle) that interact with modified head groups (red circle) of liposome phospholipids. Bilayer permeabilization is assayed by the acquisition of calcein (Cn) fluorescence upon its release from liposomes induced by tBid. Right. Chemical structures of obatoclax and des-methoxy obatoclax. **b** Obatoclax binds avidly to lipid vesicles. Addition of lipid vesicles (0–40 μM) to obatoclax (0.15 μM) in buffer at 37 °C leads to rapid obatoclax partitioning into vesicle bilayers and enhancement of obatoclax fluorescence (λ_ex/_λ_em_ = 540/575 nm, slitwidths = 10/10 nm); obatoclax half-maximally associates with bilayers at 13 ± 1 μM lipid (mean/half-range of two experiments). **c** Obatoclax transfers rapidly between distinct bilayers. Obatoclax (0.3 μM) added to lipid vesicles (10 μM) incorporating NBD-PE causes rapid energy transfer-mediated quenching of NBD-PE fluorescence (λ_ex/_λ_em_ = 470/538 nm, slitwidths = 10/10 nm) as obatoclax partitions into the vesicle bilayers (first arrow). On subsequent addition of sonicated vesicles lacking NBD-PE (20 μM; second arrow), obatoclax transfers from NBD-incorporating to NBD-PE-free vesicles, partially restoring NBD-PE fluorescence, over a time scale of seconds. **d** Proteolipsomes harboring membrane-anchored Mcl-1 and/or Bak derivatives (BakΔC*; Mcl-1ΔC*) were challenged with tBid in the presence or absence of obatoclax or Noxa BH3 peptide. Shown are representative fluorimetric assays from 3 independent experiments of calcein release from liposome in response to 40 nM tBid over time (right panel). Concentrations of assay constituents are given in the left panel

In this study, large unilamellar proteoliposomes were created that recapitulate the constitutive integral association that native Mcl-1 and Bak make with the MOM in intact cells. To that end, lipids were employed that reflect both the composition and relative abundance found in the MOM [12], but which also included low amounts of the modified lipids N-(4-maleimidobutyroyl)-PEG_3_-POPE (Mal-PEG_3_-PE) and/or the tris-(nitrilotriacetic acid)-modified lipid DOD-tris-(NTA(Ni^2+^)). Recombinant forms of human full length Bak and Mcl-1 were created in which the C-terminal TM segment was replaced with 6 His residues followed by a unique terminal Cys, and the proteins were linked to the ecto-surface of liposomes either covalently through the Cys residue (via Mal-PEG_3_-PE) or through high-affinity coordination of the His_6_ sequence to bilayer-incorporated DOD-tris-(NTA(Ni^2+^)) (Fig. 1a), thereby overcoming the otherwise difficult challenge to express and properly anchor the proteins via their native TM segment. In all experiments reported here, proteoliposomes were recovered free of unattached Mcl-1 or Bak prior to functional analyses. As reported below and in ref [1], the basic tenets that have been elucidated for the tBid - Bcl-XL - Bax model for execution and regulation of permeabilization of liposomal membranes by Bax, appear also to apply to the tBid - Mcl-1 - Bak axis, and are outlined in Fig. 1a (left).

### Obatoclax is restricted to liposomes where it is mobile

Small molecule obatoclax (Fig. 1a, right) is hydrophobic (cLogD at pH7.4 = 3.14) and insoluble in most aqueous based solvents employed for biochemical analyses of protein/small molecule interactions [14]. As predicted, obatoclax associated avidly with lipid bilayers, showing 50 % association with large unilamellar vesicles at a lipid concentration of ca. 13 μM (Fig. 1b). As illustrated in Fig. 1c, obatoclax both partitioned into lipid bilayers and transferred between bilayers with rapid kinetics (half-times <5 s for both processes at 37 °C). This suggests that, in mammalian cells in which the effective membrane lipid concentration is several mM [26], obatoclax will partition overwhelmingly into cellular membranes but also it can transfer readily between different membranes, as also suggested by cellular imaging [14].

### Obatoclax is a direct antagonist of membrane-associated Mcl-1 but not of Bcl-XL

**Fig. 2:**
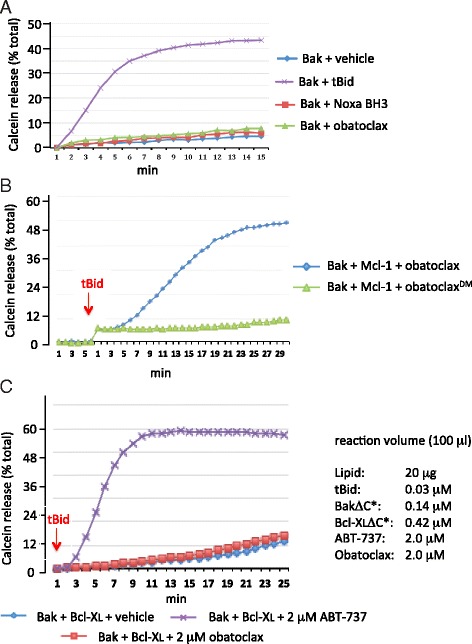
**a** Bak-dependent release of calcein from proteoliposomes is not activated by obatoclax or Noxa BH3 peptide. Assays ± tBid were conducted as described in Fig. 1d, in the presence of 1 μM obatoclax, Noxa BH3, or vehicle (0.05 % DMSO) alone. **b** As in Fig. 1d except proteoliposomes contained Bak and Mcl-1 derivatives and the assays conducted in the presence of 1 μM obatoclax or 1 μM des-methoxyl (^DM^) obatoclax. **c** As in Fig. 1d except that liposome-anchored Bcl-XL replaced Mcl-1 and ABT-737 was tested. Concentrations of assay constituents are provided in right panel. Shown are representative assays from at least 3 independent experiments

Assay mixtures (100 μl) containing proteoliposomes (20 μg lipid) with encapsulated fluorescence reporter calcein (quenched) and surface anchored Bak (0.14 μM) were challenged with 0.04 μM caspase-8-cleaved recombinant human Bid (aa 1–195 cleaved to 7 kDa and 15 kDa tBid fragments). Calcein acquires spontaneous fluorescence emission upon transbilayer release from the liposome, quantified as the % total calcein emission that is observed by treating the proteoliposomes with detergent. Conditions were selected to provide a robust Bak-dependent release of calcein in response to tBid (Figs. 1d and 2a), while at the same time minimizing the spontaneous and concentration-dependent release of calcein by Bak in the absence of tBid (Fig. 2a), which was seen at higher ratios of Bak:lipid concentration (not shown). The sub-stoichiometric ratio of tBid:Bak that was needed to observe robust release of calcein is consistent with the “hit-and-run” mechanism proposed for Bax activation in liposomes [13]. In the presence of a 3 - fold molar excess of surface-anchored Mcl-1 relative to surface-anchored Bak, the release of calcein in response to tBid was blocked (Fig. 1d).

Overcoming the ability of lipid-anchored Mcl-1 to antagonize tBid-dependent activation of Bak and release of calcein from liposomes is the predicted property of a sensitizing BH3 protein or its mimetic. Since Mcl-1 acts through binary sequestration of the activating BH3 stimulus (tBid) and of activated Bak [1], the potency of an Mcl-1 antagonist is defined by the molar excess relative to Mcl-1 that is required to overcome Mcl-1-mediated inhibition of Bak. In our assay, a 2.5 molar excess of a 25 aa peptide spanning the BH3 helix of the Mcl-1-specific sensitizing BH3 only protein Noxa or of obatoclax, relative to Mcl-1, overcame Mcl-1-mediated inhibition of tBid-induced calcein release (Fig. 1d). Since neither Noxa peptide nor obatoclax had any effect on the release of calcein from Bak-alone proteoliposomes (Fig. 2a), these agents acted by inhibiting Mcl-1 rather than by activating Bak. Moreover, under the conditions of this assay, the latter findings also indicate that neither Noxa nor obatoclax resulted in non-specific disruption of the liposomal bilayer.

Our earlier *in silico* studies of obatoclax docking into the P1 and P2 hydrophobic pockets of the BH3-binding groove of Mcl-1 predicted that the −3-methoxy moiety of obatoclax penetrated deep into the P2 pocket, driving hydrophobic binding [15]. Of note, and in contrast to obatoclax, the des- methoxy analog of obatoclax (Fig. 1a) did not overcome Mcl-1-mediated inhibition of tBid-induced, Bak-dependent release of calcein from proteoliposomes (Fig. 2b). Moreover, when assessed for cytotoxicity in KMS-11 cells, whose survival in standard cell culture depends upon Mcl-1 (see Fig. 6a), the des-methoxy analog was without toxicity compared to obatoclax (see Fig. 4a). Thus, the 3-methoxy moiety of obatoclax appears to be essential for its inhibitory activity against Mcl-1. Finally, replacing liposome-tethered Mcl-1 with liposome-tethered Bcl-XL did not allow obatoclax to overcome the inhibition by Bcl-XL of tBid-dependent Bak-mediated release of calcein, whereas the validated small molecule antagonist of Bcl-XL, ABT-737 [27], was active (Fig. 2c). This suggests that obatoclax exhibits a preference for Mcl-1 compared to Bcl-XL.

**Fig. 3:**
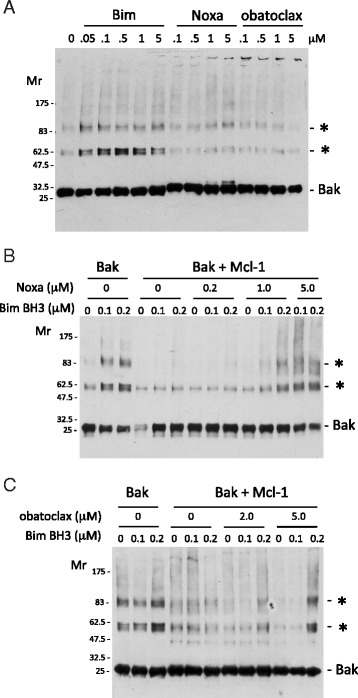
Mcl-1 inhibits Bim BH3-dependent oligomerization of Bak, which is overcome by obatoclax and Noxa BH3. **a** Bim BH3 but not Noxa BH3 nor obatoclax induce Bak oligomerization. Bak was conjugated onto liposomes and the liposomes treated with the indicated concentration of BH3 peptides or obatoclax or vehicle (1 % DMSO, lane 1). Liposome-conjugated Bak proteins were crosslinked with 0.5 mM BS^3^ and the samples analyzed by immunoblot with anti-Bak antibody. Migration of molecular weight markers is indicated. (*) denotes oligomers of Bak. **b, c** Mcl-1 (3-fold molar excess over Bak) inhibits Bim BH3-induced Bak oligomerization. Treatments and analysis were as in (**a**), in the presence of Noxa BH3 (**b**) or obatoclax (**c**) or vehicle alone, as indicated. Shown are representative blots from at least 3 independent experiments

Bak, like Bax, undergoes auto-oligomerization in the mitochondrial outer membrane in response to tBid, to form predicted transmembrane pores [28]. Employing chemical cross-linking and immunoblot, we monitored Bak oligomerization (*i.e.*, the formation of cross-linked Bak adducts) in proteoliposomes induced by a 25 aa peptide spanning the BH3 helix of the activator BH3-only protein Bim, and tested the influence of Noxa peptide and obatoclax. The results aligned well with the conclusions from the functional analyses derived by monitoring calcein release from the proteoliposomes. Bim, but neither Noxa peptide nor obatoclax, stimulated the formation of higher order Bak adducts (Fig. 3a). Excess Mcl-1 inhibited Bim-induced Bak oligomerization (Fig. 3b,c), and this inhibition was overcome by Noxa peptide (Fig. 3b) and by obatoclax (Fig. 3c).

### Mcl-1 and single agent induction of cell lethality by obatoclax *in vitro* and *in vivo*

**Fig. 4:**
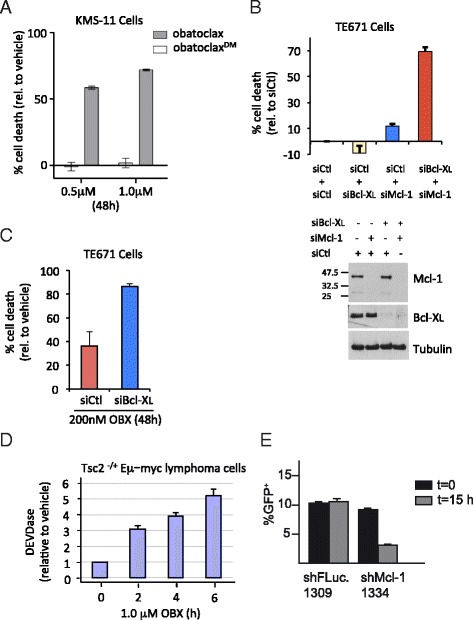
Obatoclax is active in cells whose survival depends on Mcl-1. **a** Viability of KMS-11 myeloma cells was measured in the presence of 0.5 μM and 1 μM obatoclax (gray bars) or the des-methoxy derivative of obatoclax (white bars) or vehicle (1 % DMSO) for 48 h. The % cell death was normalized to vehicle control. Error bars show the mean with SEM. **b** TE671 cells depend on both Mcl-1 and Bcl-X_L_ for survival. Cells were transfected with the indicated combinations of siRNA for 48 h. Cell death was determined and expressed as percentage of cells treated with control siRNA (siCtl) (upper panel). Western blot analysis of the siRNA treated cells (lower panel). **c** Knocking down of Bcl-X_L_ enhances death of TE671 cells by obatoclax. TE671 cells were transfected with control siRNA or Bcl-X_L_ siRNA for 24 h followed by 48-hr treatment with 200 nM obatoclax. Cell death was determined and expressed as percentage of transfected cells treated with DMSO. **d** Obatoclax induced caspase activation in Tsc2^+/−^*Eμ-Myc* lymphoma cells. Cells were treated with 1 μM obatoclax for the indicated time and DEVDase activity was determined. **e** Flow cytometry analysis of GFP-positive Tsc2^+/−^*Eμ-Myc* lymphoma cells as readout for cell survival of cells transduced with shFLuc.1309 or shMcl-1.1334. Cells were transduced once with the indicated constructs and flow cytometry analysis performed 12 h after transduction (t = 0) as well as 15 h later (t = 15). Error bars indicate SEM (n = 3)

As outlined above, the multiple myeloma cell line KMS-11 spontaneously undergoes cell death upon siRNA-mediated knock-down of Mcl-1 (see Fig. 6a). These cells also exhibit single agent lethality in response to obatoclax (Fig. 4a). In contrast, the osteosarcoma cell line T671 depends upon both Mcl-1 and Bcl-XL to confer cell survival since knock down of both proteins, but not of either protein alone, was required to induce cell death (Fig. 4b). Similarly, a combination of Bcl-XL knock down and obatoclax treatment was more lethal than obatoclax alone in these cells (Fig. 4c), consistent with the findings from liposomes that Bcl-XL is relatively more resistant to obatoclax inhibition (Fig. 2c). As a preliminary experiment to examining the single agent activity of obatoclax *in vivo*, we also examined murine Tsc2^+/−^*Eμ-Myc* lymphoma cells for their response to obatoclax treatment (Fig. 4d) or Mcl-1 knock-down (Fig. 4e) *in vitro*, both of which indicated that these cells depend on Mcl-1 for survival and, therefore, would be expected to be susceptible to single-agent obatoclax *in vivo*.

**Fig. 5:**
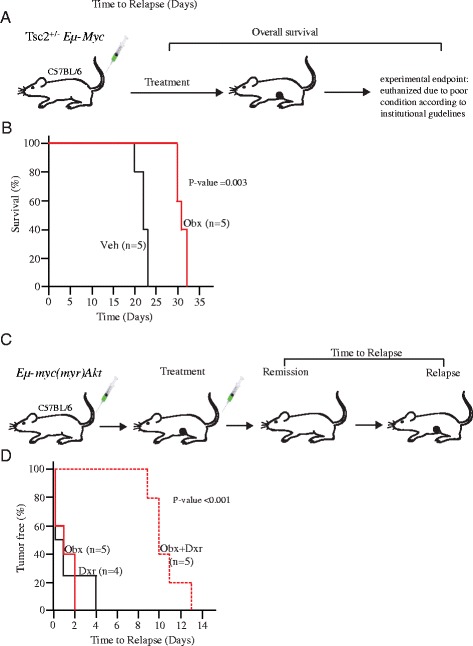
**a** Model of lymphomagenesis and treatment response. C57BL/6 mice were tail vein injected with 10^6^ Tsc2^+/−^*Eμ-Myc* lymphoma cells. Two days later, animals were randomly grouped and treatments started. **b** Kaplan-Meier plot showing overall survival of mice bearing Tsc2^+/−^*Eμ-Myc* tumors following treatment with vehicle (Veh, solid black line; *n* = 5), or obatoclax (Obx, solid red line; *n* = 5). **c** Model of lymphomagenesis and treatment response. C57BL/6 mice were tail vein injected with 10^6^ *Eμ-Myc/*(myr)Akt lymphoma cells. Upon appearance of tumors, animals were randomly grouped and treatments started. **d** Kaplan-Meier plot showing tumor free survival of mice bearing *Eμ-Myc/*(myr)Akt tumors following treatment with doxorubicin (Dxr, solid black line; *n* = 4), obatoclax (Obx, solid red line; *n* = 5), or obatoclax and doxorubicin (Obx + Dxr, dashed red line; *n* = 5)

To that end, Tsc2^+/−^*Eμ-Myc* lymphoma cells were tail-vein injected into C57BL/6 mice, which generate a well-studied murine model of B-cell lymphoma (Fig. 5a) [24]. Two days later, the mice were treated daily x 5 with either vehicle or vehicle containing obatoclax. The latter group exhibited a median survival of 31 days *vs* 22 days for the control group (*p* = 0.003) (Fig. 5b), consistent with the ability of obatoclax to overcome Mcl-1-mediated tumor cell survival in a physiological setting. In the *Eμ-myc* (myr)Akt murine lymphoma model (Fig. 5c), over-expression of Mcl-1 has previously been shown to confer resistance to the chemotherapy agent doxorubicin [24, 25]. In mice harboring this model, a combination of doxorubicin and obatoclax significantly extended tumor-free survival compared to either drug alone (Fig. 5d), again consistent with the ability of Mcl-1 inhibition to overcome resistance to doxorubicin.

### Induction of Bim sensitizes KMS-11 cells to a subsequent exposure to obatoclax

**Fig. 6:**
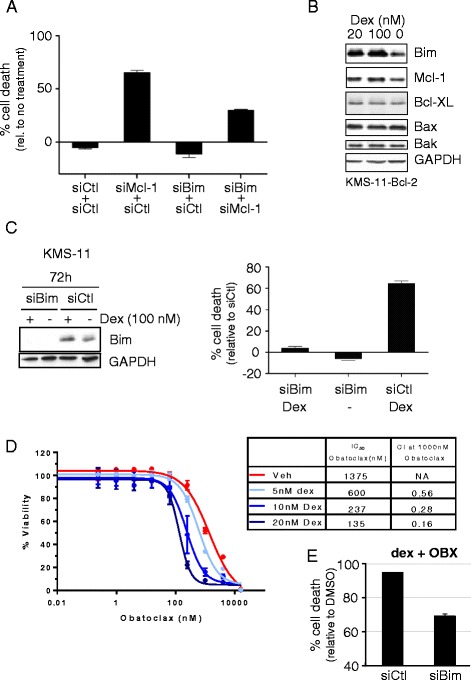
**a** KMS-11 cells were treated with the indicated combinations of siRNA. After 24 h, the percent cell death was determined and normalized to cells not treated with siRNA. Error bars show the mean of triplicate samples with SEM. **b** Western blot analysis of KMS-11 cells (expressing Bcl-2 to prevent death) treated with the indicated concentrations of dexamethasone (dex) in the presence of 40 μM zVAD-fmk for 48 h. **c** Western blot analysis of KMS-11 cells treated with Bim siRNA (siBim) or control (scrambled non-targeting) siRNA (siCtl) in the presence or absence of 100 nM dexamethasone for 72 h, and probed with antibodies against Bim and GAPDH (left panel). Cell death normalized to control siRNA (siCtl) was determined on the same samples (right panel). Error bars show the mean with SEM. **d** KMS-11 cells were pre-treated with dex for 72 h followed by exposure to obatoclax for 48 h. Viability for the combination treatments is expressed relative to samples without obatoclax in each dex dose group. Error bars represent the SD of triplicate samples. The table shows combination indices (CI) at the indicated concentrations of dex, calculated using COMPUSYN V.1.0 software according to the original method from Chou and Talalay [23]. **e** As in (D) except that viability was determined in the presence of control (siCtl) or siBim

Knock down of Mcl-1 in KMS-11 cells resulted in cell death, which could at least partly be rescued by simultaneous knock down of Bim (Fig. 6a). Thus, the survival of KMS-11 cells is determined in part by the functional ratio of Mcl-1 and Bim. One way in which this ratio can be adjusted is by treatment of KMS11 cells with dexamethasone, which increases the steady-state levels of Bim protein (Fig. 6b). Remarkably, knock down of Bim by siRNA strongly inhibited cell lethality in response to high concentrations (100 nM) of dexamethasone (Fig. 6c), indicating that Bim is a major driver of cell death in this system. Since much lower concentrations (20 nM) of dexamethasone also induce Bim and since obatoclax inhibits Mcl-1, the synthetic lethal functional relationship between Mcl-1 and Bim should also be modulated by a combination of low-dose dexamethasone and obatoclax treatment. Indeed, pretreating cells with doses up to 20 nM dexamethasone (3 days) to induce Bim (Fig. 6b) strongly reduced the IC_50_ of a subsequent dose of obatoclax (Fig. 6d), presumably because less inhibition of Mcl-1 is required to kill cells in the face of elevated Bim levels. Moreover, calculation of combination indices (CI) [23] indicated a strong synergistic relationship between obatoclax and dexamethasone (CI < 0.7) (Fig. 6d). Finally, cell lethality in response to the drug combination could at least partly be rescued by knock down of Bim (Fig. 6e), similar to the rescue of KMS-11 lethality in response to Mcl-1 knock down (Fig. 6a).

## Conclusions

Although obatoclax was originally developed as a pan Bcl-2 antagonist [14] and has reached late stage clinical development, its hydrophobic properties have precluded detailed studies of drug-target interactions in aqueous-based solutions. Employing synthetic proteoliposomes, however, we now demonstrate that obatoclax completely partitions into even relatively low concentrations of bilayer lipid, where it is a highly dynamic molecule capable of rapid exchange between lipid bilayers. Thus, obatoclax is primarily available only to targets that are associated with membrane. In the tBid-Mcl-1-Bak axis of MOMP, for example, both Mcl-1 and Bak are constitutively bound to the mitochondrial outer membrane. Employing proteoliposomes that recapitulate this topology, we show that obatoclax is a potent and direct antagonist of Mcl-1 but not of Bcl-XL. This latter observation may explain why obatoclax lacks associated thrombocytopenia in patients treated with the drug [29], a common side effect associated with the Bcl-XL antagonist navitoclax [30]. Moreover, a lipid-associated but highly mobile molecule like obatoclax may have a kinetic advantage in its ability to inhibit a Bcl-2 member such as Mcl-1, which in contrast to Bcl-XL is mostly bound to membrane in cells [1]. Finally, the chemical structure of obatoclax includes a pyrrolylpyrromethene core that in certain environments can potentially generate reactive oxygen species when bound by copper [31] and, in a protonated form, may function as a chloride ion symporter [32]. Such properties may confer cytotoxic properties to obatoclax in addition to Mcl-1 inhibition and might explain why this molecule can kill Bax/Bak-null cells in certain contexts [33]. Identifying variants of obatoclax lacking these activities may yield important new second-generation Mcl-1 lead inhibitors [34] while retaining the attractive cellular and *in vivo* activities of this class of Mcl-1 antagonist.

To that end, we also demonstrate favorable single agent activity of obatoclax in cell lines and models of murine lymphoma *in vitro* and *in vivo*, which correlate with Mcl-1-dependent cell survival. Although Mcl-1 has emerged as a significant cancer cell survival and therapy resistance factor across multiple cancer indications, it is now widely recognized that capturing the therapeutic benefit of Mcl-1 antagonism will likely rely on rational synthetic lethal strategies [1]. In the present study, a surprisingly simple example of this has emerged. Treatment of multiple myeloma KMS11 cells with dexamethasone resulted in the induction of Bim as an essential element of dex-induced cell lethality, while at the same time causing a significant reduction in the IC_50_ of subsequent obatoclax treatment. By simultaneously increasing Bim via dexamethasone and inhibiting functional Mcl-1 via clinically relevant doses of obatoclax, the Mcl-1 “rheostat” now favors cell death. Since glucocorticoids are part of most regimens used to treat multiple myeloma, the results suggest a strong rationale for also incorporating obatoclax with dexamethasone/prednisone as a clinical test of this concept.

## Abbreviations

dex: Dexamethasone
dxr: Doxorubicin
MOM: Mitochondrial outer membrane
MOMP: MOM permeabilization
OBX: obatoclax
